# Scaling-up a new socio-mental health service model in Iran to reduce burden of neuropsychiatric disorders: an economic evaluation study

**DOI:** 10.1186/s13033-021-00468-w

**Published:** 2021-05-20

**Authors:** Seyede Sedighe Hosseini Jebeli, Aziz Rezapour, Ahmad Hajebi, Maziar Moradi-Lakeh, Behzad Damari

**Affiliations:** 1 Department of Health Economics, School of Health Management and Information Sciences, Iran University of Medical Sciences, Tehran, Iran; 2 Health Management and Economics Research Centre, School of Health Management and Information Sciences, Iran University of Medical Sciences, Tehran, Iran; 3 Research Center for Addiction and Risky Behavior (ReCARB), Psychiatric Department, Iran University of Medical Sciences, Tehran, Iran; 4 Department of Community and Family Medicine, Preventive Medicine and Public Health Research Center, Psychosocial Health Research Institute, Iran University of Medical Sciences, Tehran, Iran; 5 Neuroscience Institute, Tehran University of Medical Sciences, Tehran, Iran

**Keywords:** Mental health, Economic evaluation, Universal health coverage

## Abstract

**Background:**

The integration of core packages of mental health care into routine primary health care has been introduced as an effective way to achieve universal health coverage in mental health care. Based on the transition of mental health care in Iran, from introducing basic mental health care in PHC to the experience of community-based mental health centers for urban areas, a new socio-mental health service model has been so far proposed. This study aimed to estimate the impact of scaling-up the new socio-mental health model at the national level as well as its associated costs.

**Methods:**

This study was a cost-consequence analysis following One Health Tool methodology. The data required for the study were collected in the first quarter of the year 2020 with a time horizon from 2020 to 2030. The selected metric for summarizing health effects is healthy life years gained. Resources used in terms of drug and supply, staff salaries and outpatient visits were documented and associated costs were subsequently estimated in order to estimate the average cost of each intervention per case.

**Results:**

The health impacts are calculated in terms of healthy life years gained for 20202030, after adjusting the prevalence and incidence rates for each disorder. In total, 1,702,755 healthy life years were expected to be gained. Considering total 1,363,581,654 US dollars cost in base case scenario, each healthy life years gained will cost around 801 US dollars. Based on the WHO criteria for cost-effectiveness threshold, all of the values ranged from 724 to 1119 US dollars obtained through eight different scenarios were considered as cost-effective given the GDP per capita of 5550 US dollars for Iran in 2018.

**Conclusions:**

Mental health budget in Iran equals to about three percent of total health expenditure while the mental health cost per capita is estimated to be 1.73 US dollar which are relatively low considering the share of the MNS disorders in the national burden of diseases. The results of current study showing the cost of 16.4 US dollar per capita for scaling up this comprehensive mental health service model can convince high-level policy-makers to increase the share of mental health budget accordingly. The present study demonstrated that the cost in this new socio-mental services model is not substantial compared with GDP per capita of Iran.

## Introduction

Mental health is regarded as an integral part of an individuals capacity to lead a fulfilling life. Disturbances of a persons mental well-being can thus adversely compromise this capacity as well as choices made in this respect, resulting not only in diminished functioning at individual level but also broader welfare losses for households and society [1].

Although effective interventions have been thus far introduced and affordable methods for their delivery have been shown to work, scaling-up the quality of mental health services has not occurred in most countries. Accordingly, mental health care should be included as an essential component of universal health coverage (UHC) and even fully integrated into global response to other health priorities whereas access to quality of care and financial risk protection is ensured [2].

To note, depression and anxiety are also responsible for more than 10% of the global burden of disease (GBD) and cost $1 trillion every year in terms of lost productivity. This is accordingly a significant cost with regard to the years of (healthy) life lost (YLL) and the existing situation makes life much harder for the most vulnerable people in the world. Three quarters of the mental health disease burden is observed in low-to-middle-income countries (LMICs), and governments and householdsthose least able to afford itbear the burden of mental health care costs [3].

Current coverage of essential mental health care services in LMICs is very limited. Resources made available by governments for provision of community-based and person-centered mental health care services are often very modest. The resources that are made available are also typically directed towards more specialized and institutional services, which are not easily accessible. Without appropriate access to decent services and adequate protection, individuals with mental disorders and their families are correspondingly facing a difficult choice, namely, paying out of pocket for treatments of variable and sometimes poor quality or going without treatments altogether [4].

The economic consequences of low investments in mental health are staggering, with an estimated loss of US $16 trillion to the global economy due to mental disorders (for the period: 20102030), driven in part by the early age of onset and loss of productivity across the life course. In 2011, the Grand Challenges in Global Mental Health initiative led by the United States (US) National Institute of Mental Health (NIMH) prioritized implementation research questions to reduce treatment gaps for mental disorders. The first priority identified was the integration of core packages of mental health care services into routine primary health care (PHC) ones [2].

Addressing the large and growing burden of mental, neurological, and substance use (MNS) disorders at the population level via scaled-up implementation of evidence-based treatment and prevention has been repeatedly essential over the past decade, and it can be expected to place new resource demands on the health systems of LMICs [4].

In Iran, about three percent of health care expenditure is allocated to mental health [5]. There are 20 mental health workers per 100,000 Iranians as for 2017 which has a significant increase compared to 11 per 100,000 population in 2011. The breakdown according to profession is as follows: psychiatrists (2.2 per 100,000), child psychiatrists (0.15 per 100,000), other medical doctors, not specialized in psychiatry (0.63 per 100,000), nurses (9.45 per 100,000), psychologists (5.17 per 100,000), social workers (1.51 per 100,000), occupational therapists (1.01 per 100,000) and speech therapist (0.63 per 100,000). Nearly 5192 in 100,000 of the general population visit outpatient facilities, mostly community-based facilities, while there are about 28.5 beds per 100,000 populations, in various settings including community-based facilities and hospitals [6].

Based on the transition of mental health care in Iran within about 2 decades, from introducing basic mental health care in PHC to the experience of community-based mental health centers (CMHC) for urban areas [7], a new socio-mental health service model, with two basic and advanced service strata, has been so far proposed. The given model has been piloted in eight cities in this country over the last 5years.

The first mental health program was piloted from 1992 to 1994 and after more than two decades, it now covers 18 million (82.8%) rural residents and 10 million (21.7%) citizens in urban areas. The program mainly addressed severe mental health disorders, epilepsy, and mental retardation. While it has been proven successful for villages, this program is not sufficient for those residing in urban areas. In response to the need of urban dwellers, the structure of CMHC for urban areas was designed to target those with neurotic disorders including depression and anxiety as well as severe mental disorders such as schizophrenia, bipolar disorder, and suicide attempts [8].

The new socio-mental health service model is thus comprised of two levels of health services, i.e., basic and advanced mental health services. The basic level services include public education on the basics of socio-mental health skills and screening. At this level, cases with target mental conditions are recognized by community health workers and then referred to mental health specialists for further evaluations and interventions. Using an inter-sectorial approach, patients in need of social services should be referred to a relevant service provided by other organizations in order to strengthen their social support [8].

The advanced socio-mental health services take account of facilities for mental and social emergencies and provide treatments for referrals from level one. Such services should be delivered by a collaborative care team comprised of psychologists, clinical psychiatrists, etc. at the integrated mental health settings such as PHC health centers, CMHCs, and hospitals [8].

Accordingly, scaling-up mental health services in a successful manner involves putting a range of human, physical, and other resource inputs together in order to deliver interventions and services capable of improving mental health status and related outcomes. In view of that, an essential element of evidence-based mental health service planning and scaling-up is related to an assessment of what resources are required for delivering of services to populations in need and meeting program goals [9].

Therefore, this study aimed to estimate the impact of scaling-up the new socio-mental health model at the national level as well as its associated costs to make an investment case to be considered by policy-makers for further evidence-based policy-making. The perspective chosen for this study is healthcare system, specifically ministry of health which responsible for providing mental health services through primary healthcare settings and hospitals. While healthcare perspective provides a straightforward analysis of costs and health gains for policy makers, the societal view can capture both costs and impacts in a comprehensive scale which will be more convincing once the evidence is use for budget negotiation at national level. Although, considering social costs is often in favor of cost-effectiveness of mental health interventions [10], the societal perspective has not been taken into account as the OHT methodology does not provide technical capacity for such estimation.

## Methodology

### Study design and tools

This study was a cost-effectiveness analysis to examine the expected health gains of scaling-up certain interventions as well as their costs to reduce the burden of mental disorders in different groups of patients suffering from anxiety, depression, bipolar disorder, and epilepsy in Iran. The scaling-up scenario was thus tested against the no scaling-up scenario with the existing level of care offered in the system. The data required for the study were collected in the first quarter of the year 2020 and the time horizon was a 10-year period from 2020 to 2030. Although the amount of services provided in the private sector is considerable and provides 75% of outpatient services [11], in this study we assumed ministry of health as the single provider in order to restrict the scope of research and gather high quality data to make precise estimations. It was therefore assumed that all the services were being provided by the government within the facilities of the Ministry of Health and Medical Education (MOHME) and the analysis was fulfilled based on its perspective.

### One health tool module on MNS disorders

The OneHealth Tool (OHT), a software tool developed by the international costing experts from the World Health Organization (WHO) and other United Nations (UN) agencies, was employed to start this study. The mental health module of the OHT was accordingly developed in order to ensure that the national mental health development plans have been carried out within a framework of assessment of overall health system capacity and to take financial sustainability and outcomes-based planning into account [12].

### Research planning

Consulting with three main national planners at the Department of Mental Health in the MOHME who are in charge of this program, priority MNS disorders including major depression, anxiety, bipolar disorder, and epilepsy were selected.

The main criteria to select these interventions include: (1) Provision of care within the centers affiliated by ministry of health: for instance, the drug abuse treatment was excluded as the services are mainly provided in other sectors; (2) Burden of diseases: as reflected in the introduction, these disorders are among most prevalent ones in national and global level; and (3) The inclusion of the interventions in OHT impact module: Since not all of the interventions are included in this module to estimate health impacts, we checked the available interventions and excluded ones which are not provided in impact modules and finalized eight interventions.

It is noteworthy that these interventions are recommended in WHO Mental Health Gap Action Program (mhGAP) guideline [13] as standard and effective interventions. So, their effectiveness is proved through RCTs and Systematic reviews which are formulated in the OHT to estimate the health impacts. Although national estimation of effectiveness would better describe the situation but it hasnt been in the scope of this study to estimate these measures and no national studies were available. Therefore, we relied on the global and regional evidence of effectiveness in the OHT.

Moreover, appropriate mental health care packages and scenarios, current and target coverage levels for specific intervention strategies, and scaling-up period were identified through consultations with the expert group of national planners and program managers.

For clinical-level consideration of resource use profiles for different disorders and interventions, unit costs and prices for health care services and commodities (such as those for staff salaries, outpatient visits, and psychotropic medications), one of the pilot sites (namely, the city of Oskoo in East Azerbaijan Province) was selected. This new service model was initially implemented in three cities [14]. Although a formal evaluation was not performed to compare the process in these three pilot sites, based on the regular observations, national managers of the program believe that Oskoo is the best practice to the program, benefiting from excellent documentation and assessments. To collect these data inputs, the research team, working with local team members and other national staff, identified and utilized local data sources and visited the site in a 3-day tour. The data checklist adapted from the study by Chisholm [12] was also modified and used in order to facilitate and document the process of data contextualization.

### Data contextualization

#### Health impacts

The selected metric for summarizing health effects at the population level is (healthy) life years (LYs) gained [equivalent to disability-adjusted life years (DALYs) averted], where one DALY could be thought of as one YLL. (Healthy) LYs is also computed with reference to country-specific life tables that have been already built into the model, and reflect the combined time spent by the population in a particular state of health with a known degree (or free) of disability. Disability levels were drawn from the GBD 2010 study [15]. Implementing or scaling-up an effective intervention in the population was thus modeled to reduce the time spent in a disabling state, either by reducing prevalence (e.g., by decreasing the number of new cases or increasing the remission rate), or by improving the level of functioning in people with the condition in question [12].

#### Epidemiology

Iran has a history in mental health surveys and it is therefore possible to make use of high-quality epidemiological data from the national surveys [16,18]. However, to meet age-stratified data sheets of the OHT on the prevalence and incidence rates for all disorders, the 2017 GBD estimates available in GBD result tool (http://ghdx.healthdata.org/gbd-results-tool) were used. The prevalence and incidence rates were extracted for the four disorders including depression, anxiety, bipolar and epilepsy and the data were sorted for male and female in 17 age groups. Then, to meet the OHT requirements for data entry, these rates were modified to cases per 1,000 population.

#### Estimating resources, costs, and coverage levels

The key categories of health service costs in the OHT included drug and supply costs (e.g., daily dose of a generically produced first-line anti-psychotic or anti-epileptic medication), costs of response to ambulatory contacts by mental health or general health workers (such as psychologists, counselors, and community health workers), and costs of hospital-based outpatient/inpatient care services. In addition, program-level resource needs were identified, including overall program management and administration as well as training [12].

#### Intervention costing

Resources used in terms of drug and supply, staff salaries, and outpatient visits were documented through field visits to the pilot site and the associated costs were subsequently estimated based on accounting documents available in the program management office in order to estimate the average cost of each intervention per case.

As described in the introduction of the new care model, basic package of care (e.g., basic psychosocial treatment plus medication therapy of moderate cases of anxiety/depression) were being provided in health care facilities and severe cases of those disorders in addition to all bipolar cases could be referred to the CMHCs for intensive care and social support. It is noteworthy that the WHO mhGAP guideline was practiced with slight changes in real time spent and availability of recommended drugs in the whole system of integrated mental health care in PHC.

All three levels of care [health care facility, CMHC, and general hospital (psychiatric ward)] were accordingly visited and all staff including community health workers, socio-mental health experts, general practitioners, psychologists, psychiatrists, and social workers were interviewed in order to identify activities and estimate time spent for each patient and intervention. The drug and supply list used for each intervention was also documented and the price list was acquired from the health information system (HIS) of the hospital with a particular focus on most-prescribed medications available in the primary care setting. It should be noted that drug and supply prices have the same tariffs across the country in public hospitals and the PHC network.

#### Program costing

Program specific costs concern the cost incurred at the national and provincial level to manage and supervise the program. Information on the program-specific staff required for scaling-up were obtained from background strategic documents of the program available at the Department of Mental Health including a unit cost study of new socio-mental program, so-called SERAJ, in the year 2018 [19]. In order to estimate the cost of supervision and auditory visits as well as necessary trainings, the district managers were interviewed in the field visit and costing parameters including transportation, trainings and allowances to take part in missions were identified.

#### Total cost

The total costs of scaling-up an intervention in a given year for a country was thus derived by multiplying resource use needs by their respective unit costs to give an average cost per case. It was then multiplied by the total number of cases, expected to receive a particular intervention (given by the prevalence of the disorder multiplied by the rate of treatment coverage of specific intervention strategies in the population), that is, total cost=populationprevalence ratecoveragetreatment cost per case [12]. To estimate the costs, the expected inflation rate for the period: 20202030 was applied using extrapolation based on the actual inflation rates for 20102019. Since all the costs were calculated in Iranian Rial (IRR), to convert the costs in US $, the exchange rate for the study period was estimated through extrapolation based on the UN exchange rates for the past 10years.

#### Coverage

In order to estimate present and future coverage levels, we have conducted three rounds of panel discussions with three national policy makers and three provincial managers who have been in charge of this program at national and district levels. The first panel was conducted at the beginning of the research, the second one was just at the early beginning of data collection and the last one during the filed visit in the pilot site. All the estimations of coverage were made based on the population covered in each area and expected coverage in the future based on the available budget. In order to make sure about coverage levels, we re-checked measures before and after field visits and examined different scenarios.

The estimates of the baseline coverage were based on the coverage of health care facilities per population. Given the expected challenges of scaling-up the new model at the national level, a modest coverage target of 3040% by 2030 was set for the intensive packages of care and 6070% target coverage was considered for the basic package, which have already had 40% baseline coverage.

#### Assumptions

In this study, the volume of services provided in the private sector was not estimated. It was assumed that all the services had been provided by the government within the facilities of MOHME. Besides the base case scenario which is provided below, we examined seven others scenarios in order to provide a sensivity analysis.

#### Currencies reported

At first, all the costing parameters were estimated in IRR (Iranian Rial) as local currency values were used in the context of ongoing policy dialogue, then all the cost values were converted into the US dollars for ease of interpretation and comparison. The exchange rate was captured from the UN exchange rates in June 2020 which equals to 194,881 IRR.

In addition, the final result of cost per each health life years were also reported in Intl dollars (PPP adjusted) to provide a more realistic view while the results are compared to that of different countries. In order to convert values to Intl dollars we used the cost conversion tool developed by Campbell Collaboration (https://eppi.ioe.ac.uk/costconversion/). The underlying methods of this tool is provided in the paper published in the journal Evidence and Policy [20].

## Results

After consulting the national managers of the program, the baseline coverage levels (2020) as well as the target ones (2030), were defined for each of the interventions. Among different scaling-up patterns available in the tool including linear, S-shaped, front-loaded, and exponential, the front-loaded interpolation pattern, recommended for middle-income countries was chosen. Front loaded interpolation is basically applying a calculation pattern to reflect an assumption that scaleup happens relatively quickly (in the first years of the projection) and then coverage increases slow down. We tend to see this kind of pattern when the building blocks are in place to roll out a new service or expand an existing one. The coverage levels for all the interventions in each year are presented in Table 1.

**Table 1:** Intervention coverages (%)front loaded pattern

Scale up period	2020	2021	2022	2023	2024	2025	2026	2027	2028	2029	2030
Anxiety disorders											
Basic psychosocial treatment and anti-depressant medication for anxiety disorders	40	43.9	47.5	50.9	54.1	57.1	59.9	62.6	65.2	67.7	70
Intensive psychosocial treatment and medication therapy for anxiety disorders	1	12.6	17.3	20.4	22.6	24.4	25.8	27.1	28.2	29.1	30
Depression											
Basic psychosocial treatment and anti-depressant medication of first episode cases	40	43.0	46.0	49.0	52.0	55.0	58.0	61.0	64.0	67.0	70
Intensive psychosocial treatment and anti-depressant medication of first episode cases	1	3.9	6.8	9.7	12.6	15.5	18.4	21.3	24.2	27.1	30
Intensive psychosocial treatment and anti-depressant medication of moderate-severe cases on a maintenance basis	1	3.9	6.8	9.7	12.6	15.5	18.4	21.3	24.2	27.1	30
Bipolar disorder											
Intensive psychosocial intervention for bipolar disorder, plus mood-stabilizing medication	1	12.6	17.3	20.4	22.6	24.4	25.8	27.1	28.2	29.1	30
Epilepsy											
Basic psychosocial support, advice, and follow-up, plus anti-epileptic medication	40	43.9	47.5	50.9	54.1	57.1	59.9	62.6	65.2	67.7	70

The total cost including both intervention and program costs for all interventions selected from the module of mental and neurological disorders are illustrated in Table 2. To note, all the cost values were converted into the US dollars for ease of interpretation and comparison, but local currency values were used in the context of ongoing policy dialogue. The exchange rate was captured from the UN exchange rates in June 2020 which equals to 194,881 IRR.

**Table 2:** Total costs

	2020	2021	2022	2023	2024	2025	2026	2027	2028	2029	2030	Total
Intervention costs	8,448,265	13,488,255	18,621,150	23,847,368	29,150,836	34,514,740	39,924,870	45,361,257	50,808,697	56,257,325	61,698,157	382,120,919
Program costs	141,575	301,207	424,001	560,520	683,314	819,833	942,627	1,051,697	1,162,739	1,269,838	1,378,908	8,736,258
Labor costs	31,012,194	42,009,848	53,232,422	64,666,007	76,263,942	87,984,733	99,796,025	111,651,997	123,519,471	135,378,198	147,209,639	972,724,477
Total costs	39,602,034	55,799,310	72,277,573	89,073,895	106,098,092	123,319,306	140,663,522	158,064,951	175,490,907	192,905,361	210,286,703	1,363,581,654
Per Capita	16.42											

As shown in Table 3, the health impacts are calculated in terms of (healthy) LYs gained for 20202030, after adjusting the prevalence and incidence rates for each disorder in the NCD impact module. Depression has the biggest share in the health impact, as it is the most prevalent mental disorder in Iran. In total, **1,702,755** (healthy) LYs were expected to be gained via scaling-up the program at national levels. Considering the total **1,363,581,654** US dollars cost, each (healthy) LY gained will cost around **801** US dollars.

**Table 3:** Healthy years gained

Impact scale up	2020	2021	2022	2023	2024	2025	2026	2027	2028	2029	2030	Total
Depression	0	35,873	59,717	78,750	95,490	110,979	125,688	139,854	153,611	167,043	180,231	1,147,236
Anxiety	0	8235	13,966	19,595	25,477	31,699	38,272	45,177	52,380	59,847	67,544	362,192
Bipolar	0	4461	6371	7658	8660	9498	10,229	10,884	11,480	12,027	12,534	93,802
Epilepsy	0	1691	3423	5199	7018	8879	10,774	12,698	14,645	16,610	18,588	99,525
Total	0	50,260	83,477	111,202	136,645	161,055	184,963	208,613	232,116	255,527	278,897	1,702,755

In order to provide a sensivity analysis, we have examined three sets of scenarios changing the scale up patterns, investigating the lower and upper bound of Confidence Intervals (CIs) for prevalence and incidence rates and finally changing the coverage levels.

As shown in the Table 4, the cost per a healthy LY changes from 1199 US $ in exponential pattern to 959 in linear one. Based on the health system development level in Iran, the front-loaded pattern, recommended for middle income countries, is selected and other scenarios are tested against it.

**Table 4:** Sensivity analysis

Scenarios	Base case (front loaded)	Scale up pattern		Epidemiologic data CIs		Expected coverage levels		
		Exponential	Linear	Lower	Upper	Coverage 1	Coverage 2	Coverage 3
Intervention costs	382,120,919	390,611,079	387,772,875	305,783,940	476,662,810	392,627,220	448,247,779	372,911,819
Program costs	8,736,258	8,736,258	8,736,258	8,736,258	8,736,258	8,736,258	8,736,258	8,736,258
Labor costs	972,724,477	994,357,774	987,228,401	778,324,069	1,213,512,516	1,014,058,490	1,065,250,321	934,160,222
Total costs	1,363,581,654	1,393,705,112	1,383,737,535	1,092,844,267	1,698,911,584	1,415,421,968	1,522,234,357	1,315,808,299
Impact scale up								
Depression	1,147,236	753,131	957,688	934,294	1,403,864	1,105,159	1,439,832	1,105,371
Anxiety	362,192	282,307	324,450	316,006	418,867	310,894	435,672	311,346
Bipolar	93,802	38,658	67,133	65,494	130,647	93,802	128,165	93,802
Epilepsy	99,525	88,300	94,216	61,126	150,542	65,234	99,525	65,716
Total impact	1,702,755	1,162,396	1,443,487	1,376,920	2,103,920	1,575,089	2,103,194	1,576,235
Cost per a healthy LY (US $)	801	1199	959	794	807	899	724	835
Cost per a healthy LY (Intl $/PPP adjusted)	7714	11,548	9236	7647	7772	8658	6973	8042

Then, in the next set of scenarios, the epidemiological data, obtained from GBD estimates, were changed using the CIs of reported prevalence and incidence rates resulting in a range of 794 to 807 US dollars per each healthy LY gained.

Finally, we have examined different coverage levels as the main driver for costs and impacts. In the scenario Coverage 1, we have changed the existing coverage of basic interventions from 40 to 50% while in the Coverage 2 scenario the target level for intensive care was adjusted for 40% instead of 30% in base case scenario. Moreover, in the scenario Coverage 3 the target level for the basic package were reduced considering 60% level of coverage.

Based on the WHO criteria [21], all of these values ranged from 724 to 1199 US $ obtained through eight different scenarios were considered as cost-effective.

## Discussion

In the face of large and increasing burden of mental diseases and treatment gaps existing in Iran, a new socio-mental health service model was introduced to the mental health system of this country. This program, available in PHC settings, could significantly contribute to achieving the goals of the UHC for the MNS disorders in terms of financial protection and service provision. This study was further concerned with informing national policy-makers about resource needs and costs of scaling-up such mental health services in national level.

The OHT methodology used by Chisholm [12] was followed in this study. In comparison with their estimation for five LMICs, the results of this study indicated that the resource needs for scaling-up mental health services did not need to be substantial. Although differences in health system development, service packages, and costing requirements make it much difficult to compare results between various countries in such studies, the cost estimated here for Iran in the base case scenario was 801 US dollars in terms of cost per (healthy) LY gained. This value was far less than what was estimated for South Africa, around 18,000 US dollars, whose per capita income was comparable to that of Iran as a middle-income country. This estimation was somehow analogous to that of India with 761 US dollars per (healthy) LY gained from scaling-up their mental health program [12].

In another cost-effectiveness analysis of an essential mental health intervention package in Nigeria [22], the cost per each DALY averted for a package of care including depression, schizophrenia, epilepsy, and hazardous alcohol use was estimated 320 US dollars. In addition, the study of cost-effectiveness of the Mental Health and Development model for schizophrenia-spectrum and bipolar disorders in rural Kenya [23] estimated the cost of a DALY averted around Int $727 from the health system perspectives.

In a more recent study in Ethiopia, a cost-effectiveness analysis about scaling-up essential neuropsychiatric services for treatments of depression, schizophrenia, bipolar disorder and epilepsy were performed [24]. Based on the results of this study, treatment of epilepsy with a first-generation antiepileptic drug is the most cost-effective treatment (US $321 per DALY adverted). Treatments for depression have mid-range values compared with other interventions (US $4571026 per DALY adverted). Treatments for schizophrenia and bipolar disorders are least cost-effective (US $11683739 per DALY adverted). Generally, the cost of bipolar and schizophrenia treatments are two or three times higher than that of depression and epilepsy which often dont need hospitalization and specialist services.

In the present study, through examining different scenarios, it was shown that achieving more coverage level in new socio-mental health in terms of increased level of intensive care package (increasing the expected level from 30 to 40%) could significantly reduce the cost per each (healthy) LY gained from 801 US $ in base case compared to 724 US $ in respective scenario. It means that by expanding this new service model aimed at urban population, more (healthy) LY will be gained and the cost per each (healthy) LY will decrease. Comparisons made between different scenarios show that introduction of this new service model in urban areas is cost-effective while the basic package of care is well-developed in rural areas. It is noteworthy that all the health gains based on the base case would cost 16.4 US dollar per capita in the next 10years from the health system perspective which is not substantial compared to the health expenditure per capita equal to 484 US dollars as for 2018 (https://data.worldbank.org/indicator/SH.XPD.CHEX.PC.CD?locations=IR) and can in turn greatly increase the access to the mental health care by citizens and is considered an important step forward in order to achieve universal health coverage in mental health. In addition, as reported in an economic analysis study [25] based on the unit costs of this program in the pilot level, considering 116,570 people in order to be screened and treated within this model, the per capita cost was estimated 5.56 US dollar which seems comparable to our estimation as we did a prospective estimation in the national level for the next 10years considering inflation rate.

Furthermore, based on 2014 atlas of mental health [26], the per capita mental health cost in Iran is reported 1.73 US dollars while in the same year the health expenditure per capita equals to 432 US dollars. This figure demonstrates how neglected is the mental health sector and how this new service model could contribute to greater access and improved mental health with relatively small amount of funds.

Furthermore, based on the WHO criteria for cost-effectiveness threshold [21], cost per (healthy) LY gained (i.e., DALY averted) was considered very cost-effective if it was less than a gross domestic product (GDP) per capita in a specified country. The estimation of 801 US dollars (7714 Intl dollars) per (healthy) LY gained through the scaling-up period of the new socio mental health care could be regarded cost-effective as well given the GDP per capita of 5550 US dollars (12,937 Intl dollars) for Iran in 2018 (2019) (https://data.worldbank.org/indicator/NY.GDP.PCAP.PP.CD?locations=IR).

Therefore, the health returns on such an investment are substantial. Such information on the costs and health impacts of scaling-up could further provide important evidence that could be used in dialogues with health planners and policy-makers at the national level, particularly in the context of increased policy attention to the rising burden of mental and neurological disorders.

The estimates reported herein additionally represented an initial set of projections, based on national available evidence and informed inputs of local experts; however, such estimates are subjected to further discussions, reviews, and revisions as planning cycles and political processes are evolving. New projections will be accordingly prepared in the light of changes to policies and plans, such as revised target coverage levels or lengthened implementation periods.

## Conclusion

Mental health budget in Iran equals to about three percent of total health expenditure while the mental health cost per capita is estimated to be 1.73 US dollar [5, 26], which are relatively low considering the share of the MNS disorders in the national burden of diseases. The results of current study showing the cost of 16.4 US dollar for scaling up this comprehensive mental health service model besides previous studies (19, 25) reporting 5.56 US dollar per each person, can convince high-level policy-makers to increase the share of mental health budget accordingly. Using the OHT strategic planning reflecting on the expected health gains and the associated costs, could help policy-makers with evidence-informed policy- making to prioritize cost-effective interventions and to improve allocative efficiency. The present study provided a timely analysis as the Department of Mental Health in Iran sought to implement an ambitious plan of mental health scaling-up across the country, collaborating with the MOHME and the Ministry of Interior. Therefore, this work could make a useful contribution to state-level deliberations on the implementation of this plan at national level. Expanding such works beyond the mental health to all non-communicable diseases (NCDs) and making comparisons within and between specific packages of cares can be thus great evidence while the programs are developing at different departments aimed to be scaled-up at national and sub-national levels.

## Limitations

Working with the OHT through different modules can be a challenging job with hundreds of parameters to be revised and updated. A broad concern is also related to the available national evidence on a number of domains. These include epidemiological data on the burden of the MNS disorders, extent of current coverage and expenditure, as well as evidence base for locally adapted cost-effective interventions. While regional and global default estimates are available, the tool developers strongly encourage updating parameters with national estimates.

To update the prevalence and incidence rates, the GBD estimates were accordingly used as the last national mental health survey of Iran did not include estimates on the incidence rates while the prevalence rates had been also limited to major depression and anxiety. In case of cost-effectiveness of interventions and transition rates (e.g., remission and mortality rates), the default OHT rates were employed, since there were no national estimates or even studies investigating such parameters.

## Data Availability

The datasets used and/or analyzed during the current study are available from the corresponding author on reasonable request.

## Abbreviations

UHC: Universal health coverage
YLL: Years of (healthy) life lost
GBD: Global Burden of disease
LMICs: Low and middle income countries
US: United States
NIMH: National Institute of Mental Health
PHC: Primary health care
MNS: Mental, neurological, and substance use
CMHC: Community-based mental health centers
MOHME: Ministry of Health and Medical Education
OHT: One health tool
WHO: World Health Organization
UN: United Nations
DALYs: Disability-adjusted life years
mhGAP: Mental health gap action program
HIS: Health information system
CIs: Confidence intervals
NCDs: Non-communicable diseases
